# Quantitative assessment of lumbar intervertebral disc degeneration and its correlation with clinical symptoms: a study utilizing ultrashort time-of-echo and T2 mapping as biomarkers

**DOI:** 10.1186/s12891-025-09039-3

**Published:** 2025-09-01

**Authors:** Jun Han, Xiang Hong Meng, Zhilin Ji, Feige Niu, Nana Zhu, Tingting Zhao, Zhiwei Shen, Zhi Wang, Qiang Yang

**Affiliations:** 1 The Department of Radiology, Tianjin Hospital, Tianjin University, 406 Jiefang Southern Road, Tianjin, China; 2 Philips healthcare, Tianzhe Road 16, Chaoyang, Beijing, China; 3 The Department of Spine Surgery, Tianjin Hospital, Tianjin University, 406 Jiefang Southern Road, Tianjin, China

**Keywords:** Intervertebral disc degeneration, Cartilage endplate, T2 mapping, Ultrashort echo time T2* mapping

## Abstract

**Background:**

Lumbar cartilage endplate (CEP) structures show low signal intensity on conventional magnetic resonance imaging (MRI), making them hard to observe and quantify. This often results in poor correlation between conventional MRI findings and low back pain (LBP) symptoms and provides inadequate guidance for clinical decisions.

**Methods:**

The study included Twenty-five healthy volunteers and forty-one patients with LBP. Quantitative MRI techniques—Ultrashort Echo Time (UTE) T2* mapping and T2 mapping are employed to evaluate lumbar intervertebral disc degeneration (IVDD) and LBP symptoms. Pfirrmann and Rajasekaran grading systems and the Oswestry Disability Index (ODI) served as reference standards.

Regions of interest (ROIs) for the nucleus pulposus (NP), upper CEP, and lower CEP were outlined in UTE Two echo subtracting images and transferred to UTE images fused with 3D water sequence images and T2 mapping images. UTE-T2* and T2 mapping values were automatically calculated. Cohen’s kappa, Spearman’s rank correlation, and Kruskal–Wallis tests were used, with significance set at *p* < 0.05.

**Results:**

Spearman’s rank correlation revealed that UTE‑T2* and T2 values for upper CEP, lower CEP, and NP negatively correlated with Pfirrmann and Rajasekaran grades (*P* < 0.001). The Kruskal–Wallis test showed significant differences in values between Pfirrmann grades II, III, IV, and V (*P* < 0.001). ODI was negatively correlated with T2* and T2 values of the lower CEP (*P* < 0.001) and positively with Pfirrmann grade (*r* = 0.2,*P* = 0.003).

**Conclusion:**

Quantitative MRI methods for T2* values and T2 mapping are associated with grade of degeneration and ODI index and are more effective for assessing CEP damage and LBP symptoms than conventional MRI sequence.

## Introduction

Low back pain (LBP) is a leading cause of disability [1]. Research indicates that approximately 70–85% of individuals worldwide will experience symptoms of LBP at some point in their lives [2]. In the United States, the annual cost of LBP exceeds $100 billion, with two-thirds of these expenses being indirect due to lost wages and reduced productivity [3]. Notably, fewer than 5% of individuals with LBP account for 75% of these costs [4]. Consequently, identifying effective strategies for preventing LBP and mitigating its impacts is crucial. LBP can arise from various conditions, including trauma, metabolic diseases, benign and malignant tumors, and spinal degenerative diseases, with intervertebral disc degeneration (IVDD) being a predominant cause [5].

The intervertebral disc (IVD) consists of a central nucleus pulposus (NP), an outer annulus fibrosus (AF), and a cartilage endplate (CEP) that covers the upper and lower surfaces [6]. The CEP is a hyaline cartilage layer ranging from 0.1 to 1.6 mm and is vital for maintaining the disc’s hydrostatic pressure and facilitating nutrient transport, given the disc’s lack of direct blood supply [7, 8]. With age, the CEP undergoes structural and compositional changes, including tissue disorders, hypertrophy, thinning, defects, ectopic calcification, glycosaminoglycan (GAG) reduction, and increased collagen X [9]. These alterations can impair nutrient diffusion to disc cells, leading to decreased hydrostatic pressure, NP dehydration, and deterioration in IVD structural and mechanical properties, contributing to IVDD progression [10, 11]. Therefore, assessing CEP changes is crucial for evaluating disc degeneration [12].

Magnetic resonance imaging (MRI) is commonly used for lumbar imaging due to its detailed depiction of IVD structure, good tissue contrast, and absence of radiation. Traditional MRI grading systems, such as the Pfirrmann grade [13] and Modic [14] grades, primarily assess disc degeneration based on morphological structure and signal strength, often overlooking CEP morphology. The Rajasekaran grading system [15], which classifies endplates into six grades based on damage severity, offers a precise evaluation of CEP changes and can aid in clinical treatment decisions. However, conventional MRI sequences typically detect only changes in disc morphology and fail to capture biochemical components.

Conventional MRI sequences struggle to display CEP tissue due to the rapid signal decay from its ultrashort T2 relaxation time [16]. Research [17] indicates that the T2 relaxation times of the cartilaginous endplate typically measure less than 40 ms. Conversely, the standard Echo Time (TE) for T2-weighted imaging (T2WI) sequences generally falls within the range of 50 to 100 ms. This discrepancy results in the endplate exhibiting minimal visibility on T2WI, where it characteristically appears as a hypointense area with low signal intensity. Consequently, this inherent low signal poses significant challenges for the accurate assessment of endplate pathologies.

Recent advances, such as ultra-short echo time (UTE) MRI, have addressed this limitation by employing semi-excitation pulse and radial signal sampling to reduce echo times to less than 1 ms. These techniques enable visualization of short T2 tissues, including CEP, osteochondral junctions, and bone cortices, which are often invisible in traditional MRI sequences [18]. UTE quantitative techniques, such as UTE T1rho and T2*values, can quantitatively assess biochemical changes in the IVD and the degree of degeneration at the molecular level [19].

Furthermore, traditional degenerative grading standards based on T2- weighted (T2W) and T1-weighted (T1W) MRI sequences may not strongly correlate with patients’ symptoms [20]. MRI findings have not consistently predicted the development or duration of LBP [21]. Tools, such as the Oswestry Disability Index (ODI) [22] and Japanese Orthopaedic Association (JOA) scores [23], are frequently used to assess LBP symptoms, but some studies have found weak correlations among these scores, MRI findings, and LBP symptoms [24, 25]. This disconnect may arise from conventional MRI sequence’s inability to detect subtle tissue changes that are relevant to LBP symptoms and clinical decision-making.

The objective of this study was to evaluate the feasibility of using UTE T2* and T2 values to assess CEP damage in individuals with chronic low back pain (CLBP). We analyzed the relationship among these quantitative MRI values, clinical LBP symptom scores, and grading systems (Pfirrmann and Rajasekaran grade) to determine whether MRI quantitative value measures can serve as objective standards for evaluating disc degeneration grade and associated clinical symptoms.

## Materials and methods

### Participants

This study received approval from the ethics committee of Tianjin University, Tianjin Hospital under project number “2023 Medical Review (Medical ethics) 158.” All participants provided written informed consent and our study is in accordance with the Declaration of Helsinki.

This study included 66 volunteers and patients with symptoms of LBP who underwent lumbar spine MRI at Tianjin University, Tianjin Hospital between July 2023 and January 2024. Inclusion criteria were: Aged 20–65 years, encompassing healthy volunteers and individuals with low back pain (LBP). Participants reporting LBP symptoms exhibited chronic low back pain persisting for at least six months prior to the current MRI examination. Furthermore, these individuals had undergone a lumbar MRI scan within the preceding six-month period, which demonstrated the presence of degenerative changes in their lumbar intervertebral discs.

**Fig. 1:**
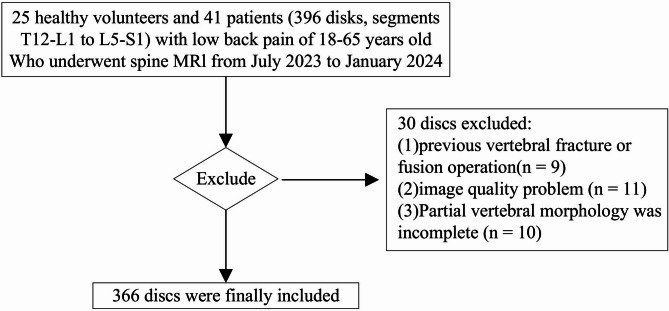
Inclusion and exclusion criteria

Exclusion criteria included a history of spinal fracture, spinal tumors, metastases, tuberculosis or other infectious diseases, lumbar surgery, and contraindications to MRI. A detailed inclusion and exclusion flowchart is presented in Fig. 1. Participant demographics, including age and sex, were recorded. Information on chronic lower back pain (occurring daily for the past year) and the ODI was collected for all participants.

### MRI acquisition

Lumbar spine scans were conducted using an 8-channel 3.0T MRI Scanner (Ingenia CX, Philips Healthcare, Best, Netherlands). Examination sequences included T1-weighted imaging (T1WI), T2-weighted imaging (T2WI), ultrashort echo time (UTE),3D Water, and T2 mapping sequences, covering the T12-S1 vertebra.

The multi-echo UTE sequence used parameters based on 3D fast field echo sequences, employing short rectangular hard excitation pulses and acquiring data filled in a three-dimensional radial pattern from the center of the K-space outward in a conical shape. Scans were performed with participants in a supine position, with the head positioned first. Detailed parameters for each scan sequence are provided in Table 1.

**Table 1:** MRI protocol for imaging of the lumbar

Sequence	UTE-T2* mapping	T2 mapping	3D WATS	T1 WI	T2 WI
Orientation	Sagittal	Sagittal	Sagittal	Sagittal	Sagittal
Fat Suppression	SPAIR	NO	NO	NO	SPAIR
TR (ms)	10	2000	2000	450	1600
TE(ms)	0.2,3.8,7.5	13; 26; 39; 52; 65; 78	13	9	65
FOV(mm)	200 × 200	212 × 212	200 × 200	170 × 170	153 × 153
matrix	256 × 256	256 × 256	256 × 256	256 × 192	256 × 192
Slice Thickness(mm)	4.5	4.5	4.5	3	3
Flip Angle (°)	3°	90°	15°	90°	90
NEX	1	1	1	1	1
Acquisition Time	8 min 15 s	4 min 5 s	1 min	2 min	1 min 8s

*SPAIR* spectral attenuation with inversion recovery, *WI* weighted imaging, *PD* proton density, *TR* repetition time, *TE* echo time, *FOV* field of view, *NEX* number of excitations

### Image analysis

**Fig. 2:**
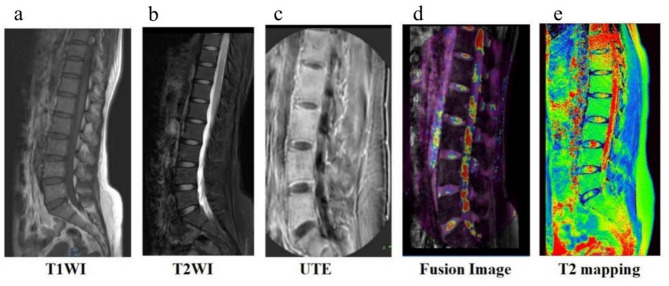
Magnetic resonance images of different sequences. From a woman who underwent a lumbar MRI examination, 28 years old, no history of low back pain, **a**. T1WI sequence image, **b**. T2WI sequence image, **c**. UTE sequence echo 1 image, **d**. UTE subtraction sequence and 3D water sequence overlay image, **e**. T2 mapping sequence image

**Fig. 3:**
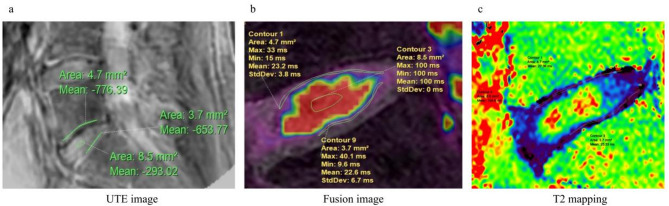
ROI sketching. From the L5-S1 vertebrae of a woman who underwent a lumbar MRI examination, 28 years old, no history of low back pain. **a** UTE Different echo subtraction image, the ROI initially outlined contains: contour 1, the area of the upper endplate, had an area of 4.7mm^2^; contour 3, the region of the nucleus pulposus, with an area of 8.5mm^2^; contour 9, the area of the lower endplate, has an area of 3.7mm^2^. The sketched ROI is then pasted into the fusion image and T2mapping image. **b** UTE subtraction sequence and 3D water sequence overlay image, The ROI copied from figure a contains: contour 1, an average UTE-T2 * value of 23.2ms; contour 3, with an average UTE-T2 * value of 100ms; contour 9, has an average UTE-T2 * value of 22.6ms. **c** T2 mapping sequence image, The ROI location and area outlined are consistent with those in a; contour 1, a mean T2 value of 27.36ms; contour 4, a mean T2 value of 104.6ms; contour 3, an average T2 value of 25.33ms

The fusion of UTE sequence and 3D Water sequence images was used to display the structure of each part of the IVDs, facilitating region-of-interest (ROI) sketching. The images of each sequence are shown in Fig. 2. The NP and CEP were identified in the UTE Two echo subtracting images, respectively. For the delineation of the regions of interest (ROIs), the anatomy-adaptive method was primarily utilized.The NP was identified as a circular area of approximately 8–10 mm^2^ at the center of the disc, while the CEPs appeared as highly linear signaling areas above and below the disc. The scope of these structures was determined using the software’s sketching tool. Outlined areas were then transferred to the corresponding location in the UTE and 3D Water fusion images and T2 mapping image. Image registration ensured alignment between the UTE and T2 mapping images. The ROI sketching area is shown in Fig. 3. The post-processing platform (Philips IntelliSpace^®^ PACS) automatically calculated T2* and T2 mapping values. Measurements were taken from three consecutive middle layers, and the average was used as the final result. UTE-T2* values were calculated using a mono-exponential fitting with custom code in Matlab (MathWorks, Natick, MA).

Disc degeneration was categorized using Pfirrmann grades (five categories) and Rajasekaran grades (six categories). Evaluation of all IVDs and area delineation was completed by two musculoskeletal radiologists (JZL and MXH, with 2 and 15 years of musculoskeletal diagnostic experience, respectively) to assess inter-observer consistency. Both radiologists performed measurements at three levels and averaged the results. T2WI was used to assign Pfirrmann and Rajasekaran grades. In cases of disagreement, the radiologists consulted each other to reach a final diagnosis.

### Statistical analysis

Statistical analyses were performed using SPSS software (version 22.0; IBM, Armonk, NY, USA) and GraphPad Prism (version 9.0; GraphPad Software, San Diego, CA, USA). Cohen’s kappa test and intraclass correlation coefficient (ICC) assessed interobserver agreement in evaluating IVDD and quantitative values for each IVD part. Spearman’s rank correlation analyzed the relationships between UTE T2* and T2 values with Pfirrmann and Rajasekaran grades, age, and sex, and between T2* and T2 values. Additionally, Spearman’s rank correlation assessed the relationships between ODI scores and UTE T2* and T2 values, as well as Pfirrmann and Rajasekaran grades. The independent sample Kruskal–Wallis test compared T2* and T2 values of lumbar IVDs across different levels and grades. Statistical significance was set at *p* < 0.05.

## Results

### Characteristics of research participants and images

This study involved 66 participants. The demographic information of the subjects is shown in Table 2. We examined six IVDs per participant, spanning from T12/L1-L5/S1. Thirty discs were excluded due to poor image quality or prior spinal surgery, resulting in a final total of 366 IVDs for analysis.

**Table 2:** Demographics of the subjects

Number	Subject					
	Total	Male	Female			
	66	40	26			
Number	Total Age			Male Age		Female Age
	33.7 ± 12.9 years (range, 20–65 years)			33.2 ± 12.9years		34.7 ± 13.2years
Number	Pfirrmann grade					
	I	II	III	IV	V	
	52	217	74	18	5	
Number	Rajasekaran grade					
	I	II	III	IV	V	VI
	9	168	126	34	19	10

**Fig. 4:**
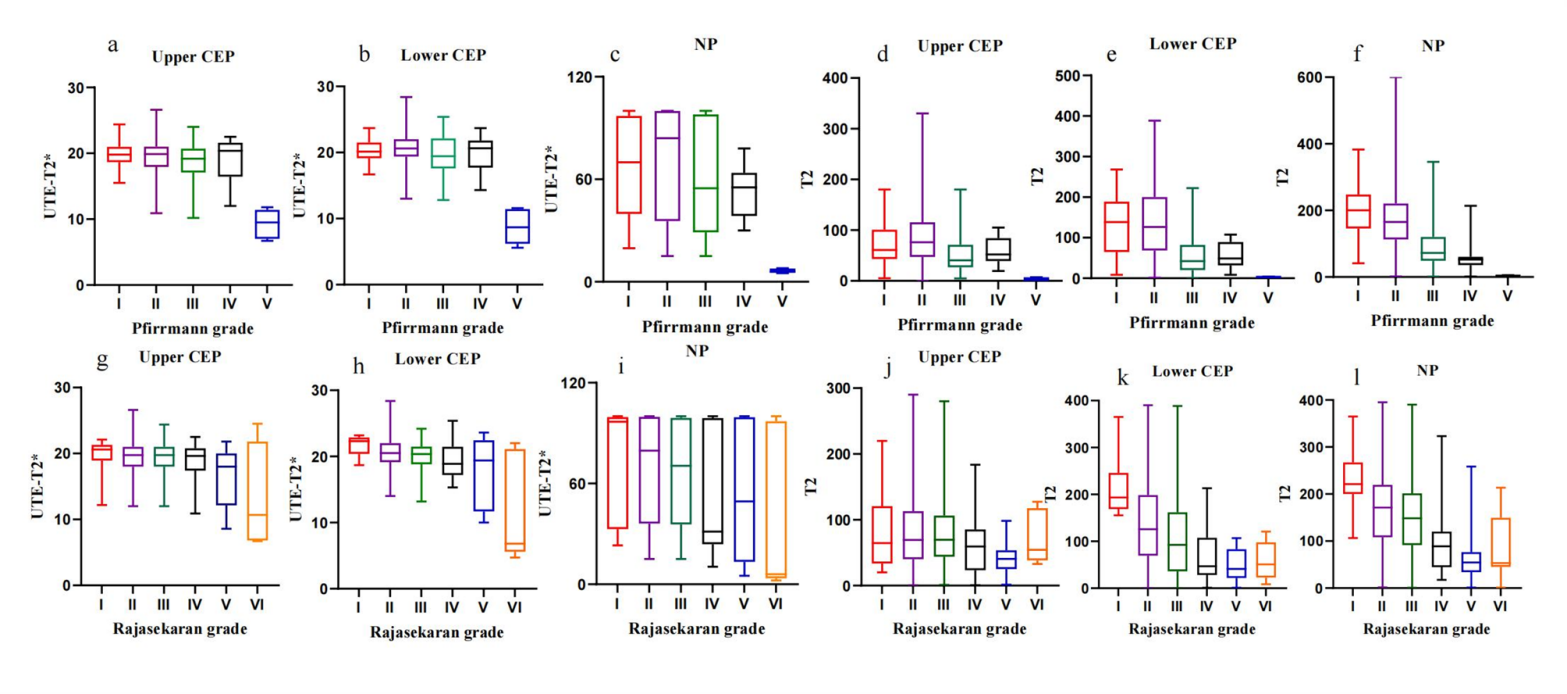
Box plot of the distribution of UTE-T2* values and T2 values in different Pfirrmann and Rajasekaran grades. **a**, **b**, **c** and **g**, **h**, **i** are respectively UTE‑T2* relaxation time of upper CEP, lower CEP and NP; **d**, **e**, **f** and **j**, **k**, **l** are respectively T2 value of upper CEP, lower CEP and NP. NP Nucleus pulposus, UTE Ultrashort time-to-echo, CEP cartilage endplate

Figures 4 illustrates the distribution of UTE T2* values and T2 values across each Pfirrmann and Rajasekaran grade. The inter-observer agreement for Pfirrmann and Rajasekaran grading was substantial, with Cohen’s kappa values of 0.78 and 0.79 (*P* < 0.001), respectively. The inter-observer agreement for the quantitative IVD values was also substantial, with an ICC of 0.76 (*P* < 0.001).

### Correlations among quantified values, IVD degeneration, and post hoc multiple comparisons

**Fig. 5:**
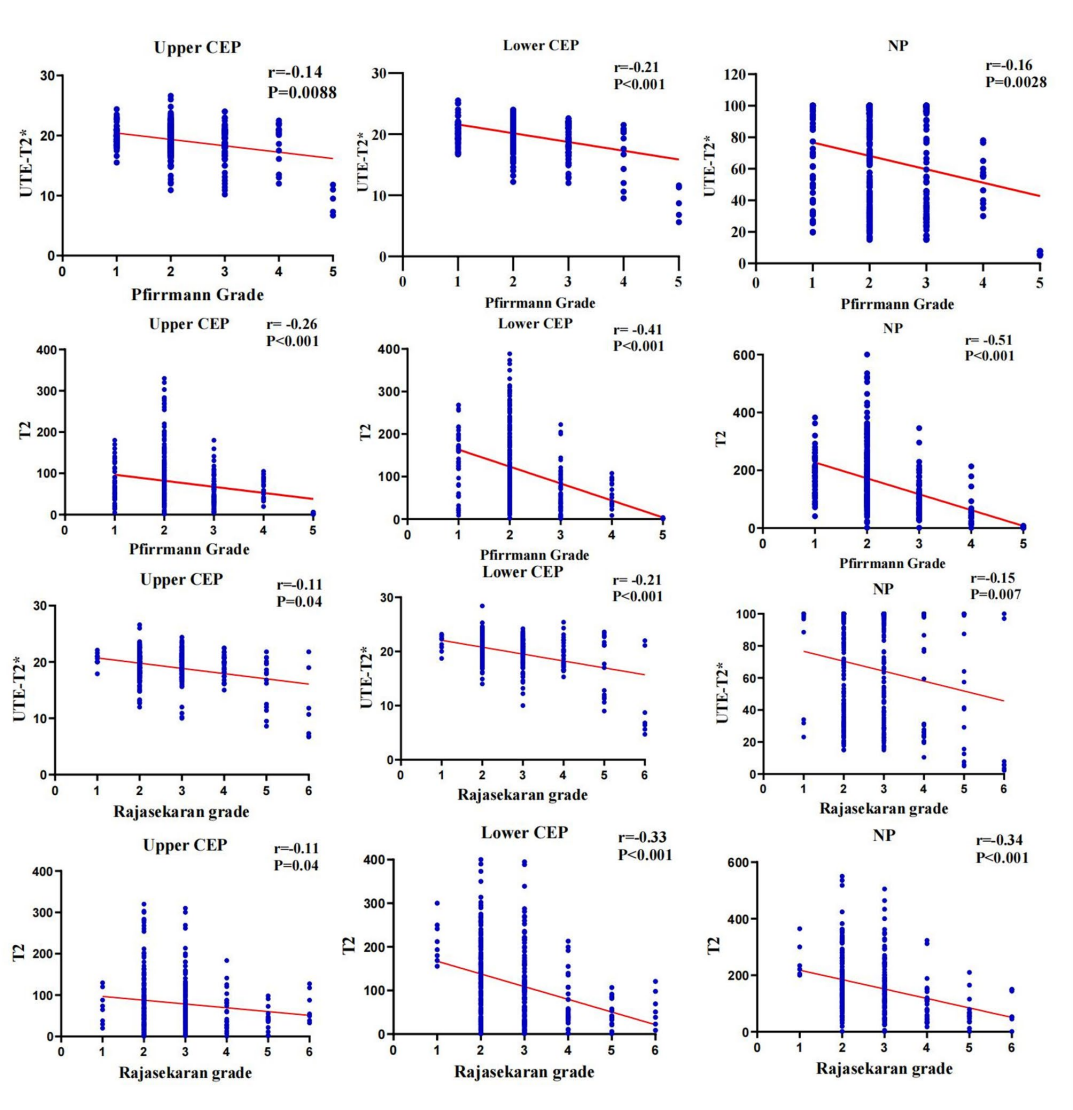
Scatter plots of the values in upper CEP, lower CEP and NP by the Pfirrmann grades and Rajasekaran grades. The T2* values and T2 values at the three sites exhibited a negative correlation with the Pfirrmann and Rajasekaran grades, **a**, **b**, **c** and **g**, **h**, **i** are respectively UTE‑T2* relaxation time of upper CEP, lower CEP and NP correlated with disc degeneration grading; **d**, **e**, **f** and **j**, **k**, **l** are respectively T2 value of upper CEP, lower CEP and NP correlated with disc degeneration grading. NP Nucleus pulposus, UTE Ultrashort time-to-echo, CEP cartilage endplate

Spearman’s correlation analysis revealed positive correlations between UTE T2* values of the upper and lower endplates and NP with T2 values. Conversely, UTE T2* and T2 values of these regions were negatively correlated with Pfirrmann grades, Rajasekaran grade, disc segment, and participant age but were not significantly related to the participant’s sex (*P* = 0.65). Correlation coefficients and P value are detailed in Table 2. The correlations are also summarized in Fig. 5.

**Fig. 6:**
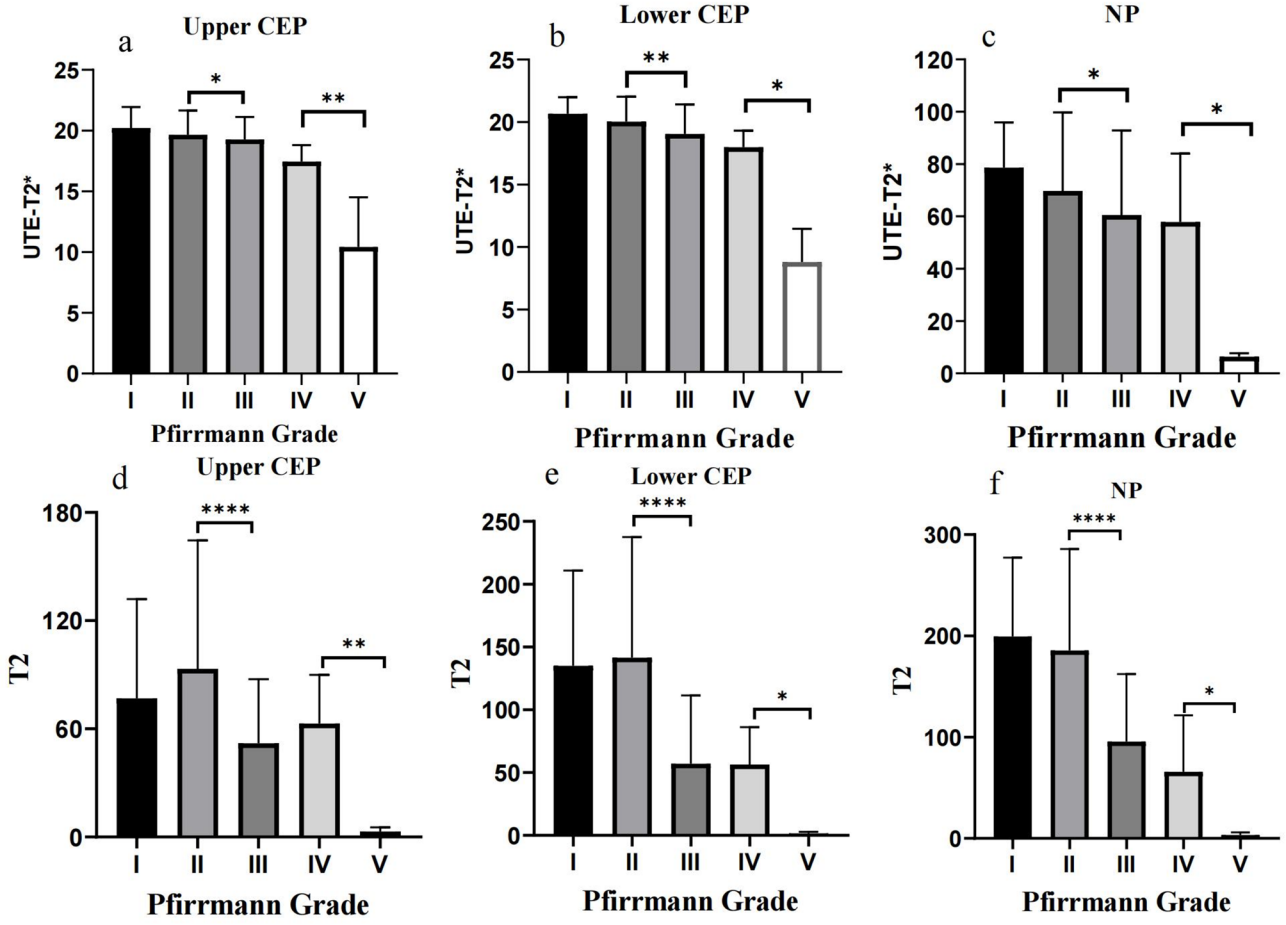
Post hoc multiple comparisons among 5 Pfirrmann grades. Significant differences were observed in UTE‑T2* values of NP and PAF between Pfirrmann grades II and III as well as between grades IV and V grades. T2 values also showed significant differences between Pfirrmann grades II and III, and between grades IV and V, in upper CEP, lower CEP, and NP. *P values of < 0.05, **P values of < 0.01, ***P values of < 0.001, ****P values of < 0.0001 were considered statistically significant. NP Nucleus pulposus, UTE Ultrashort time-to-echo, CEP cartilage endplate

The Kruskal–Wallis test indicated statistically significant differences in UTE-T2* values and T2 values only between Pfirrmann grades II and III and between grades IV and V. No significant differences were observed between neighboring Rajasekaran grades. Results are summarized in Fig. 6.

### Correlations between MRI characterization and ODI index

Spearman correlation analysis found no correlation between ODI scores and T2* or T2 values of the upper endplate and NP. However, ODI scores were negatively correlated with T2* and T2 values of the lower endplate and the average quantitative value of the lower endplate (*20.09 ± 5.11ms*,*118.49 ± 65.13ms*) was higher than that of the lower endplate (*19.06 ± 3.15ms*,*80.31 ± 50.14ms*). The correlation coefficients and P-values are provided in Table 3. There was no significant correlation between ODI scores and Rajasekaran grades (*P* = 0.72). ODI scores were positively correlated with Pfirrmann grade (*r* = 0.2,*P* = 0.003).

**Table 3:** The relationship between the quantitative values of different parts of the disc, and the correlation between the quantitative values and the grade of degeneration, segment, age, and clinical symptoms

*R* value		Pfirrmann grade	Rajasekaran grade	Disk segment	Age	T2* value	ODI index
Upper CEP	UTE-T2*	−0.14(*P* = 0.0088)	−0.11(*P* = 0.04)	−0.12(*P* = 0.01)	−0.1(*P* = 0.0135)		
	T2	−0.26(*P* < 0.001)	−0.11(*P* = 0.04)	−0.16(*P* = 0.0024)	−0.26(*P* < 0.001)	0.23(*P* < 0.001)	
Lower CEP	UTE-T2*	−0.21(*P* < 0.001)	−0.21(*P* < 0.001)	−0.34(*P* < 0.001)	−0.1(*P* = 0.0135)		−0.125(*P* = 0.024)
	T2	−0.41(*P* < 0.001)	−0.33(*P* < 0.001)	−0.51(*P* < 0.001)	−0.26(*P* < 0.001)	0.17(*P* = 0.001)	−0.212(*P* = 0.004)
NP	UTE-T2*	−0.16(*P* = 0.0028)	−0.15(*P* = 0.0074)	−0.11(*P* = 0.04)	−0.21(*P* < 0.001)		
	T2	−0.51(*P* < 0.001)	−0.34(*P* < 0.001)	−0.23(*P* < 0.001)	−0.54(*P* < 0.001)	0.12(*P* = 0.02)	

*NP* Nucleus pulposus, *UTE* Ultrashort time-to-echo, *CEP* cartilage endplate, *R* value represents the correlation coefficient of spearman correlation analysis corresponding to the two values

## Discussion

This study utilized UTE MRI, T2 mapping, and conventional MRI sequences to quantitatively measure the upper and lower endplates and NP of T12-S1 IVD and to assess degeneration grades. These findings showed negative correlations between UTE T2* and T2 values and the Pfirrmann and Rajasekaran grades, disc segment, and participants’ age. These correlations can help evaluate the reliability of T2* and T2 values for diagnosing disc degeneration and assess whether T2* values serve as objective criteria for IVDD. The ODI was negatively correlated with the lower endplate’s quantitative values and positively correlated with the degeneration grade, suggesting that quantitative measures can aid in diagnosing LBP.

Disc degeneration results from changes in various disc components. Aaron Jet al. (18) found that UTE T2* values correlated well with Pfirrmann grades and collagen contents in cadaveric IVDs. Similarly, T2 values have been shown to correlate with the composition and degeneration of lumbar discs [26, 27], though most studies focus on T2 mapping and T2* mapping with limited research on UTE-T2* mapping. This study explored the relationship between quantitative values and degeneration and their interactions.

Our results indicated that UTE T2* and T2 values of the endplates and NP were correlated with degeneration grades, with T2 values showing a stronger correlation. The correlation between UTE-quantified T2* and T2 values demonstrates consistency in reflecting disc degeneration trends. Wu et al. [28]also reported negative UTE T2* and T2 values and degeneration grades, though differences in research parameters and equipment may account for variations.

Quantitative values’ sensitivity to biochemical components and tissue properties differs. T2 value is more responsive to changes in water content, while T2* values are sensitive to tissue integrity, reflecting biochemical changes in the disc’s ultra structure [29, 30]. Both values indicate different aspects of disc degeneration, with water and proteoglycan content being crucial.

The study’s correlation analysis revealed that patient age and disc segment influence degeneration grades, consistent with the histological studies [12, 31]. Biomechanical studies suggest that the lumbar disc’s capacity to bear weight increases from T12 to L1, which aligns with our findings [32]. 

The comparison of quantitative values between adjacent grades revealed significant differences only between Pfirrmann grades II and III and IV and V. This is likely due to compositional changes in the disc during degeneration [33]. Grade I and II discs showed minimal changes, while grades III and IV represent major degeneration stages. Grade V discs exhibited substantial changes, explaining the significant differences observed. In contrast, adjacent Rajasekaran grades showed no significant differences, likely due to subtle variations.

Lin et al. [34]identified a correlation between low CEP T2* values and severe disc degeneration in younger individuals, while Wu et al. [28]found statistically significant differences between degeneration grades. Variations in results may be attributed to differences in patient demographics and sample sizes.

Previous studies have linked lumbar disc degeneration with LBP symptoms using UTE sequences. Pang et al. [35]found significant correlations between UTE morphological changes and poor ODI scores (*r* = 0.311; *p* = 0.001) or LBP (*p* = 0.009). Our study, focusing on the relationship between qualitative values and clinical symptoms, found that lower endplate changes were more strongly associated with LBP symptoms and the average quantitative value of the lower endplate was higher than that of the upper endplate, It is suggested that the lesion of the lower endplate may be an early factor in the occurrence of low back pain symptoms. The Rajasekaran grade did not correlate with LBP, whereas the Pfirrmann grade was positively correlated. This suggests that Pfirrmann’s grading accurately reflects the relationship between disc degeneration and LBP.

This study had several limitations. First, histological verification is lacking, necessitating further cadaveric or animal studies. The GAG and collagen content changes during disc degeneration affect biomechanical and transport properties, impacting nutrient transport and overall degeneration [36–38]. Further studies should include histological validation to analyze the relationship between quantitative values and disc components. Second, many correlation values obtained were weak, and statistical differences between adjacent grades were not significant. Disc degeneration is influenced by multiple factors, and correlation with a single factor may be insufficient. Further research is needed to validate the use of UTE and T2 mapping in clinical diagnosis. Expanding the study cohort and increasing the sample size could help ensure uniformity and reliability in the results across different degeneration grades and age groups. Third, the correlation between patient imaging parameters and the ODI for LBP symptoms was relatively low. This phenomenon primarily stems from the multifactorial etiology of LBP. Pathologies affecting the lumbar ligaments, including the ligamentum flavum and interspinous ligaments, as well as lumbar spinal stenosis and facet joint degeneration, all contribute to LBP symptomatology. Consequently, when analyzing the correlation between LBP symptoms and single-level intervertebral disc degeneration, the resultant correlation coefficients are inherently diminished. Future research necessitates comprehensive consideration of the diverse etiological factors influencing LBP to enhance the thoroughness of analytical investigations. Finally, the present study lacks longitudinal follow-up data regarding the progression of participants’ conditions. Therefore, in subsequent research, we will incorporate longitudinal follow-up data by conducting longitudinal follow-up studies. This will enable the systematic assessment of the dynamic changes in UTE-T2* and T2 mapping values over time, exploring their potential value in predicting the progression of lumbar disc degenerative disease and therapeutic response.

## Conclusion

In summary, both the UTE quantitative T2* value and the T2 mapping quantitative T2 value showed varying degrees of correlation with IVDD. Significant statistical changes in quantitative values were observed in Pfirrmann grades II and III as well as IV and V. Additionally, patients’ lower back pain symptoms correlated with changes in the quantitative values of the lower endplate and changes in the Pfirrmann grade. These findings suggest that MRI quantification of T2* values and T2 mapping T2 values could be valuable tools for assessing CEP damage and LBP symptoms in clinical settings, with the potential for further refinement in the future.

## Data Availability

Data is provided within the manuscript.

## Abbreviations

CEP: Cartilage endplate
MRI: Magnetic resonance imaging
LBP: Low back pain
UTE: Ultrashort Echo
IVDD: Intervertebral disc degeneration
ROIs: Regions of interest
NP: Nucleus pulposus
GAG: Glycosaminoglycan
T2W: T2-weighted
T1W: T1-weighted
ODI: Oswestry Disability Index
